# Dosimetric and NTCP analyses for selecting parotid gland cancer patients for proton therapy

**DOI:** 10.1177/03008916241252544

**Published:** 2024-05-21

**Authors:** Anna Maria Camarda, Maria Giulia Vincini, Stefania Russo, Stefania Comi, Francesca Emiro, Alessia Bazani, Rossana Ingargiola, Barbara Vischioni, Claudio Vecchi, Stefania Volpe, Roberto Orecchia, Barbara Alicja Jereczek-Fossa, Ester Orlandi, Daniela Alterio

**Affiliations:** 1 Radiation Oncology Unit, Clinical Department, National Center for Oncological Hadrontherapy, Pavia, Italy; 2 Division of Radiation Oncology, IEO European Institute of Oncology IRCCS, Milan, Italy; 3 Department of Oncology and Hemato-oncology, University of Milan, Milan, Italy; 4 Medical Physics Unit, Clinical Department, National Center for Oncological Hadrontherapy, Pavia, Italy; 5 Unit of Medical Physics, European Institute of Oncology IRCCS, Milan, Italy; 6 Tecnologie Avanzate Srl, Turin, Italy; 7 Scientific Directorate, European Institute of Oncology IRCCS, Milan, Italy; 8 Department of Clinical, Surgical, Diagnostic and Pediatric Sciences,University of Pavia, Italy

**Keywords:** NTCP models, parotid gland cancer, intensity modulated proton therapy, patient selection

## Abstract

**Purpose/Objective:**

To perform a dosimetric and a normal tissue complication probability (NTCP) comparison between intensity modulated proton therapy and photon volumetric modulated arc therapy in a cohort of patients with parotid gland cancers in a post-operative or radical setting.

**Materials and methods:**

From May 2011 to September 2021, 37 parotid gland cancers patients treated at two institutions were eligible. Inclusion criteria were as follows: patients aged ⩾ 18 years, diagnosis of parotid gland cancers candidate for postoperative radiotherapy or definitive radiotherapy, presence of written informed consent for the use of anonymous data for research purposes. Organs at risk (OARs) were retrospectively contoured. Target coverage goal was defined as D95 > 98%. Six NTCP models were selected. NTCP profiles were calculated for each patient using an internally-developed Python script in RayStation TPS. Average differences in NTCP between photon and proton plans were tested for significance with a two-sided Wilcoxon signed-rank test.

**Results:**

Seventy-four plans were generated. A lower Dmean to the majority of organs at risk (inner ear, cochlea, oral cavity, pharyngeal constrictor muscles, contralateral parotid and submandibular gland) was obtained with intensity modulated proton therapy vs volumetric modulated arc therapy with statistical significance (p < .05). Ten (27%) patients had a difference in NTCP (photon vs proton plans) greater than 10% for hearing loss and tinnitus: among them, seven qualified for both endpoints, two patients for hearing loss only, and one for tinnitus.

**Conclusions:**

In the current study, nearly one-third of patients resulted eligible for proton therapy and they were the most likely to benefit in terms of prevention of hearing loss and tinnitus.

## Introduction

Salivary gland cancers (SGCs) represent a rare disease, accounting for approximately 2-6.5 % of all head and neck cancers (HNCs).^1^ Parotid gland is the most common site, representing approximately 65-80% of all SGCs. Parotid gland cancers (PGCs) are characterized by wide variability in their biology, natural history and histology with the most frequent being mucoepidermoid carcinomas, adenoid cystic carcinomas and adenocarcinomas.^1^ The mainstay of treatment for resectable tumors is parotidectomy followed by postoperative radiotherapy (PORT) in high-risk patients defined at pathological report on the basis of adverse prognostic factors (T size, lymph node involvement, close/positive margins, vascular/perineural invasion, bone invasion and high grade).^2-8^ Unresectable disease and macroscopical residual after surgery are usually managed with radiotherapy (RT) alone.^1,9^

Intensity modulated radiotherapy (IMRT) and volumetric modulated arc therapy (VMAT) are commonly used for the management of PGCs, currently replacing 3D-conformal technique (3D-CRT).^9,10^ However, acute and late radiation-related toxicities are still frequent. In particular, the most frequent acute side effects include mucositis, dermatitis and otalgia, while late toxicities include trismus, xerostomia, sensori-neural hearing loss, tinnitus and skin fibrosis.^11^ Among these, impairment of acoustic structures shows a significant impact on patients’ quality of life (QoL) and represents one of the key points of cancer survivorship.^12^ Despite the modern techniques, the IMRT/VMAT dosimetric advantage alone does not seem to be enough to overcome these toxicities. As demonstrated by the COSTAR trial,^13^ a lower mean dose to the ipsilateral cochlea did not translate into a clinical advantage and IMRT did not provide any clinically-relevant reduction in hearing loss as compared to the 3D-conformal technique.

In this scenario, intensity modulated proton therapy (IMPT) represents a promising option for treatment-related morbidity reduction. Due to their physical properties, protons exhibit a peculiar depth-dose profile, characterized by the so-called Bragg peak, thus allowing optimal target coverage while minimizing dose to normal structures.^14^ Dosimetric comparison studies between IMPT and IMRT techniques have suggested that lower (mean) doses can be delivered to several organs at risk (OARs) in patients with unilateral HNCs and pediatric SGCs.^15-18^ In particular, proton therapy (PT) was associated with a favorable acute toxicity and dosimetric profile in pediatric population.^18^ The sharp dose gradient may achieve avoidance of adjacent structures such as the cochlea, the temporal lobe of the brain, the mandible or the oral cavity.^15,19^ However, there are still few clinical studies which compare proton and photon therapy in this peculiar setting. Despite these limited data, all clinical studies have shown a benefit in toxicity outcomes in favor of PT.^19-21^ Recently, a study by Hanania et al.^22^ with a median follow-up of 41 months, found that the rate of late grade 2 or higher toxicities was lower with respect to COSTAR trial in the arm of volumetric modulated arc therapy (VMAT).^13^

Despite these findings, randomized clinical trials (RCTs) to support the clinical advantages of PT compared to IMRT are lacking. Due to the higher costs and relatively limited availability of IMPT with respect to conventional photon-based RT, PT should be reserved for patients that are likely to benefit the most in terms of toxicity risk reduction. To address this issue, Langendijk et al.^23^ developed the so-called model-based approach, to identify head and neck cancer patients that will most benefit from PT in terms of expected probability of side effects from normal tissue complication probability (NTCP) models. The NTCP model-based strategy has been approved in the Netherlands and nowadays represents a feasible, practical and cost-effective tool to select patient candidates for PT.^24,25^

In order to test this promising strategy, this work aimed to compare in-silico treatment plans of patients with PGCs already treated in a post-operative or radical setting with photon or PT at two Italian Centers with the following goals: (i) perform a dosimetric comparison between VMAT and pencil beam scanning – intensity modulated proton therapy (PBS-IMPT) plans; (ii) quantify the potential benefit of PBS-IMPT vs VMAT in terms of NTCP, referring to a set of toxicities (dysphagia, dysgeusia, trismus) with a particular focus on toxicity of the inner ear (hearing impairment and tinnitus).

## Patients and methods

### Patient population

This study retrospectively compared treatment plans of patients with PGCs treated in a post-operative or radical setting with VMAT at the Radiation Oncology Department of the European Institute of Oncology (IEO in Milan, Italy) and IMPT at the National Center for Oncological Hadrontherapy (CNAO in Pavia, Italy) between May 2011 and September 2021.

Inclusions criteria were as follows: 1) Patients aged 18 years or older; 2) histological diagnosis of parotid gland tumor both benign and malignant; 3) Indication for photon or proton RT in adjuvant or definitive setting (total prescribed doses was 60 Gy (RBE) at least, 2 Gy (RBE)/fraction); 4) presence of written informed consent for the use of data in anonymous form for research purposes. Both benign and malignant histologies were included. Patients with diagnosis of adenoid cystic carcinoma or other tumor histologies with histologically or radiologically proven perineural invasion were excluded.

Thirty-seven consecutive patients with parotid gland tumors treated with post-operative or definitive RT were eligible for the analysis. Nineteen patients (51%) were treated at IEO while eighteen patients (49%) were treated at CNAO. Nine patients (24%) were treated with definitive RT and among these four patients (11%) did not undergo surgical resection due to patients’ refusal.

### Ethical aspects

The present study was approved by the Ethical Committee of the European Institute of Oncology, IEO, IRCCS (UID 2954) and all procedures performed in this study were in accordance with the 1964 Helsinki declaration and its later amendments.

### Computed tomography simulation, organs at risk and clinical target volume contouring

All patients underwent computed tomography (CT) simulation with axial images acquired at 2-2.5 mm at the two institutions. Thermoplastic masks were used for immobilization of the head, neck and shoulders. CT images were imported into RayStation treatment planning system (TPS) ver. 9B (RaySearch Laboratories, Stockholm, Sweden) for photon treatment planning and RayStation ver.10B (RaySearch Laboratories, Stockholm, Sweden) for proton treatment planning.

Contouring of OARs was performed by the same radiation oncologist, according to the international consensus guidelines for CT-based delineation of OARs in the Head & Neck (HN) region.^26^ The contouring of the inner ear was based on specific guidelines.^27^ OARs contours were not adjusted in case of overlap with target volumes, meaning that the mean dose (Dmean) parameter reflects the actual dose in the entire OAR, including the part overlapping the target. Contouring of masseter muscle was also performed. Clinical target volumes (CTV) were delineated according to COSTAR trial^13^: CTV1 included the post-operative parotid bed or residual and levels Ib-II-III lymph nodes. In the case of benign tumors (such as pleomorphic adenoma) only surgical bed was contoured with a margin of 3 mm. For patients requiring elective neck irradiation, CTV2 included Ib-V levels, according to pathological or clinical findings. The contouring of lymph nodes was based on international guidelines.^28^

For patients with gross tumor volume (GTV), the contouring was based on imaging findings, including CT and magnetic resonance imaging (MRI). For photon-based plan, a planning target volume (PTV) was obtained, representing geometrically expansion of 5 mm from CTV (according to institutional policy).

### Treatment planning

For this study purpose, two plans were provided for each patient:

- a VMAT plan, consisting of 6 MV photons, dual full coplanar arcs was done. Plans were optimized through Raystation TPS ver.9B (Raysearch laboratories AB, Stockholm, Sweden) using Collapsed Cone v 5.5. The PTV was cropped from the skin by 3 mm for planning optimization purposes and no bolus was applied.
- a pencil beam scanning IMPT plan, optimized with RayStation TPS ver. 10B with IMPT. The proton fixed beamlines available at CNAO were used, simulating a gantry geometry for plan optimization. Beam angles arrangement consisted of two coplanar fields with 55°/145° gantry for left targets and two coplanar fields with 340°/240° gantry for right parotid tumors. Lateral spot spacing was 3 mm, while energy layer spacing was 2 mm. A 3 cm thick range shifter was included in the optimization for adequate coverage of the shallower parts of CTV. The RayStation Monte Carlo dose engine was used for dose calculation with a 2 mm dose grid. Unlike the photon plans, where the PTV was used for CTV coverage purposes, a robust planning strategy based on minimax optimization^29^ was introduced for proton plans. For robust optimization, both ±3 mm setup and ±3% range uncertainties were considered. Relative biological effectiveness (RBE) was set to a constant value of 1.1 for RBE-weighted dose calculation.

A simultaneous integrated boost (SIB) strategy was used for both treatment plans. For this in-silico study, IMPT plans were optimized on the CTV following the same goals as the VMAT plans. Clinical goals were at least 95% of the CTV covered by 98% of the prescribed dose (D95 > 98%), while dose to the 2% of CTV less than 107% (D2 < 107%). OARs dose constraints followed the technical guidelines Head and Neck Cancer Working Group of the Italian Association of Radiotherapy and Clinical Oncology (AIRO).^30^

### Dosimetric analysis

D95 and D2 were considered for CTV coverage comparison. Regarding OARs, a dose-volume comparison was performed, particularly focusing on the contralateral parotid gland, ipsilateral cochlea and inner ear, ipsilateral temporal lobe, brainstem, mandible, oral cavity, ipsilateral submandibular glands, pharyngeal constrictors, supraglottic larynx, ipsilateral temporal-mandibular joints (TMJ) and masseter muscle. Wilcoxon signed-rank tests (p<.05) were performed to compare dosimetric parameters between VMAT and IMPT plans.

### NTCP-based analysis

After a literature review, six NTCP models were selected (Table S1 Online Supplementary Materials)^31-36^; all of them were photon-based, with exception of the NTCP model for hearing impairment.^34^ These NTCP models resulted equally distributed between levels 1a to 4, according to the classification for level of evidence proposed by Stieb et al.^37^ with reference to the TRIPOD statement.^38^

Following the model-based approach, NTCP profiles of VMAT and IMPT plans were calculated for each individual patient using an internally-developed Python script in RayStation TPS. We aimed at primarily quantifying the number of patients qualified for PT in terms of ∆NTCPx-p for hearing loss and tinnitus and secondarily the number of patients qualified for PT for ∆NTCPx-p mucositis, dysphagia, dysgeusia and trismus.

According to thresholds defined by the National Indication Protocol for Proton therapy (NIPP), patients could be qualified for PT in case the ΔNTCPx-p ⩾ 10% for grade ⩾ 2 and/or ⩾ 5% for grade ⩾ 3 side effects, respectively.^24^ If the difference between VMAT and IMPT corresponded with at least one of the predefined thresholds for ΔNTCP, then the patient could be considered for PT. Moreover, average differences of NTCP values between VMAT and IMPT for each endpoint (ΔNTCPx-p) were tested for significance with a two-sided Wilcoxon signed-rank test.

## Results

Patient and disease characteristics are shown in Table 1. Briefly, the most common histology was adenocarcinoma (19%), followed by mucoepidermoid carcinoma (16%).

**Table 1.** Patient and disease characteristics.

		Patients	
		n	%
Sex	Male	19	51
	Female	18	49
Age	18 - 60 years	20	54
	> 60 years	17	46
	Median	57	
Disease status	Primary	32	86
	Recurrent	5	14
Nature of disease	Malignant	32	86
	Benign	5	14
T stage	pT1-pT2	16	43
	pT3-pT4	10	27
	NA (Benign)	5	14
	cT1-T2	2	5
	cT3-T4	2	11
N stage	pNx	11	30
	pN0	10 (1)*	27
	pN1	4	11
	pN2	1	2
	cN0	6 (2)*	16
	NA (Benign)	5	14
Histology	Adenocarcinoma	7	19
	Acinic cell carcinoma	2	5
	Epithelial-myoepithelial carcinoma	1	3
	Carcinoma ex-pleomorphic adenoma	5	14
	Myoepithelial carcinoma	1	3
	Mucoepidermoid carcinoma	6 (1)**	16
	Pleomorphic adenoma	5 (4)**	14
	Oncocytic carcinoma	2	5
	Salivary duct carcinoma	2	5
	Secretory carcinoma	2 (1)**	5
	Others	3	8

Note: * R2: Macroscopic Residual. ** Recurrent disease.

A total of 74 plans were generated. CTV characteristics are summarized in Table 2.

**Table 2.** Volume characteristics.

		Patients	
		n	%
Nodal neck RT volumes	High-middle neck volume	14	38
	High-middle-low neck volume	6	16
CTV level doses	HD-CTV	6	16
	HD-CTV + ID-CTV + LD CTV	3	8
	HD-CTV+ LD-CTV	28	76
		**Median (cc)**	**Average (cc)**
Volume CTV (cc)	HD-CTV	69.25	70.9
	ID-CTV	120.86	142.6
	LD-CTV	187.82	198.6

Abbreviations: CTV: clinical target volume; HD: high dose; LD: low dose, RT: radiotherapy.

CTVs consisted of two levels of dose for most plans (76%) and an intermediate level of dose was delineated in three patients. Target volumes were defined as follows: High dose CTV (HD-CTV), Intermediate dose CTV (ID-CTV), Low dose CTV (LD-CTV). Fourteen patients (38%) had high-middle neck volumes (defined as CTV including lymph-nodes levels Ib-II-III) and six patients (16%) had high-middle-low neck volumes (defined as CTV including levels Ib-V). Seventeen patients (46%) had no prophylactic nodal volume.

A 2 Gy dose per fraction was prescribed to HD-CTV for most plans (76%), with a median prescription dose of 66 Gy. Dose prescriptions to LD-CTV ranged between 54 Gy to 58.1 Gy. Dose prescriptions to ID-CTV ranged between 63 Gy to 59.4 Gy. For proton plans dose was prescribed in Gy (RBE). Details about both dose levels and prescriptions are shown in Table 3.

**Table 3.** Prescription dose.

		Patients	
		n	%
Prescription Dose	HD-CTV 60 Gy, LD-CTV 54 Gy	8	22
	HD-CTV 66 Gy, LD-CTV 56.1 Gy	9	24
	HD-CTV 66 Gy, LD-CTV 59.4 Gy	4	11
	HD-CTV 70 Gy, ID-CTV 63 Gy, LD- CTV 58.1 Gy	2	5
	HD CTV 66 Gy, ID-CTV 59.4 Gy, LD-CTV 56.1 Gy	1	3
	HD-CTV 69.96 Gy, LD-CTV 56.1 Gy	8	22
	HD-CTV 69.96 Gy	1	3
	HD-CTV 60 Gy	2	5
	HD-CTV 66 Gy	2	5
		**Median (Gy)**	**Average (Gy)**
Prescription Dose	HD-CTV	66,00	65,69
	LD-CTV	56,10	55,72

Abbreviations: CTV: clinical target volume; HD: high dose; LD: low dose.

Average doses to 99%, 98%, 1% and 2% of the HD-CTV for IMRT and IMPT plans are shown in Table S2 (Online Supplementary Materials). In all the considered plans, 98% of the prescription dose encompassed ⩾ 95% of the target volume.

### Dosimetric comparison

**Figure 1.**
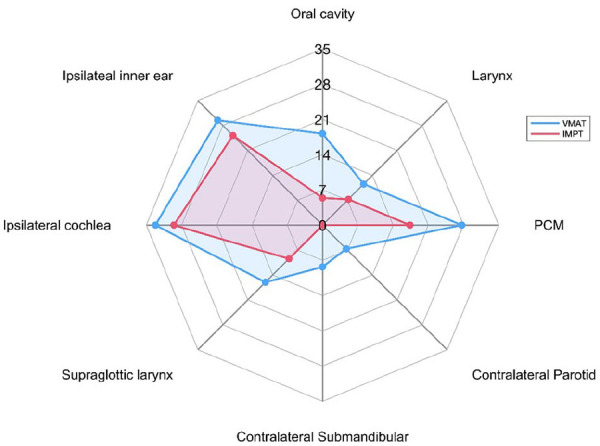
Dosimetric comparison (values displayed in Gy) between average mean doses to chosen OARs with VMAT and IMPT techniques. Abbreviations: PCM: pharyngeal constrictor muscles; IMPT: intensity modulated proton therapy; VMAT: volumetric modulated arc therapy.

Median Dmean to oral cavity, contralateral parotid gland, contralateral submandibular gland, ipsilateral cochlea, inner ear, pharyngeal constrictor muscles and larynx is shown in Figure 1 for both techniques. A statistically significant (p < .05) lower Dmean to the majority of OARs of interest, including cochlea and inner ear, was obtained with IMPT if compared to VMAT. The D1_%_ of brainstem and ipsilateral temporal lobe were also evaluated. A ΔDmean to OARs included in the dosimetric comparison, was assessed and all dosimetric results are shown in Table 4 and Figure 1.

**Table 4.** Comparison of average and median Dmean of defined organs at risk (OARs) between volumetric modulated arc therapy (VMAT) and intensity modulated proton therapy (IMPT).

Dmean of the OARs (Gy)			
		Average	Median
Oral Cavity	VMAT	18.17	17.78
	IMPT	5.40	4.48
	Δdose	12.76	12.67
	p-value *(VMAT vs. IMPT)*	**<.00001**	
Larynx	VMAT	11.56	8.78
	IMPT	7.26	4.58
	Δdose	4.3	3.48
	P value *(VMAT vs. IMPT)*	**<.00001**	
PCM	VMAT	27.64	27.97
	IMPT	17.34	17.31
	Δdose	10.30	10.43
	P value *(VMAT vs. IMPT)*	**<.00001**	
Contralateral Parotid	VMAT	6.65	6.26
	IMPT	0,00	0,00
	Δdose	6.64	6.25
	P value *(VMAT vs. IMPT)*	**<.00001**	
Contralateral Submandibular gland	VMAT	8.31	8.94
	IMPT	0.03	0
	Δdose	8.28	8.94
	P value *(VMAT vs. IMPT)*	**<.00001**	
Supraglottic Larynx	VMAT	16.05	15.95
	IMPT	9.44	7.6
	Δdose	6.61	7.41
	P value *(VMAT vs. IMPT)*	**<.00001**	
Ipsilateral Cochlea	VMAT	33.20	28.12
	IMPT	29.53	30.4
	Δdose	3.67	5.51
	P value *(VMAT vs. IMPT)*	.**01314**	
Ipsilateral Inner ear	VMAT	29.45	23.82
	IMPT	25.18	23.05
	Δdose	4.27	6.003
	P value *(VMAT vs. IMPT)*	.**00252**	

Bold values denote statistical significance at the *p* < 0.05 level.

### NTCP model comparison

Median differences between NTCP values for each of the six endpoints are reported in Table 5. Results of the trismus model by Lindbloom et al.^32^ are not shown because we obtained a NTCP close to zero for both techniques. NTCP values across all patients were not statistically different (p>0.05) between the two techniques for hearing loss and tinnitus models, whereas they were for the other 4 endpoints (Table 5). According to the NIPP ΔNTCP thresholds, among the 37 patients included in the analysis, 10 (27%) would have been qualified for PT for hearing loss and tinnitus: among them, 7 qualified for both endpoints, 2 patients qualified only for hearing loss, and 1 for tinnitus (Table S3, Online Supplementary Materials). Considering photon plans, the average NTCP value for hearing loss was 16%, while 14% considering protons, with an average ΔNTCP_x-p_ value of 2%. On the contrary, none of the patients were qualified for PT based on trismus, acute oral mucositis grade > 1.5, dysphagia and dysgeusia.

**Table 5.** The comparison of volumetric modulated arc therapy versus intensity modulated proton therapy plans and patient groups in terms of NTCP values.

		Median	Average
NTCP Physician-rated swallowing dysfunction 6 months after (CH) RT^31^	VMAT	0.03	0.03
	IMPT	0.01	0.01
	ΔNTCP		0.02
	P value (VMAT vs. IMPT)	**0.00001**	
NTCP Dysgeusia-HNQOL^33^	VMAT	0.03	0.03
	IMPT	0.01	0.01
	ΔNTCP		0.02
	P value (VMAT vs. IMPT)	**0.00001**	
NTCP Dysgeusia-UWQOL^33^	VMAT	0.03	0.03
	IMPT	0.01	0.01
	ΔNTCP		0.02
	P value (VMAT vs. IMPT)	**0.00001**	
NTCP Hearing loss34	VMAT	0.00	0.16
	IMPT	0.00	0.14
	ΔNTCP		0.02
	P value (VMAT vs. IMPT)	0.134	
NTCP Tinnitus^35^	VMAT	0.07	0.24
	IMPT	0.09	0.24
	ΔNTCP		0.00
	P value (VMAT vs. IMPT)	0.267	
NTCP Acute oral mucositis Grade>1.5^36^	VMAT	0.05	0.05
	IMPT	0.02	0.02
	ΔNTCP		0.03
	P value (*VMAT vs. IMPT*)	**0.00001**	

Abbreviations: CH: chemotherapy; IMPT: intensity modulated proton therapy; NTCP: normal tissue complication probability; PCM: pharyngeal constrictor muscles; RT: radiotherapy; VMAT: volumetric modulated arc therapy.

Bold values denote statistical significance at the *p* < 0.05 level.

## Discussion

The current work investigates whether IMPT could reduce absorbed dose to OARs compared to VMAT in patients affected by PGCs, with a particular focus on the reduction of treatment-related acoustic toxicities. Using NTCP models, our analysis showed that IMPT could have a theoretical clinical advantage in nearly one-third of the patients in our series according to NIPP criteria. To the best of our knowledge, the current study is the first to evaluate the model-based approach in PGCs.

Our findings are in line with other works which obtain similar results in multiple subsites of HNCs and nasopharyngeal cancers.^25,39^ Recently, Tambas et al.^25^ reported their first experience with model-based selection of head and neck patients for PT. Of 227 patients, 141 (62%) were qualified for plan comparison, 80 (35%) were eventually selected for PT. Of note, none of these patients had SGCs, most of them had oropharynx and laryngeal cancers which could explain why most patients were selected for PT based on the ΔNTCP for dysphagia-related toxicities.^25^

Our results also showed a dosimetric advantage on structures located in the middle-low level dose of prescription, which translated into a lower Dmean in the majority of OARs. Specifically, the oral cavity, contralateral salivary glands, pharyngeal constrictor muscles, larynx and ipsilateral temporal lobe showed statically significant differences in favor of IMPT over VMAT. These findings are consistent with recent literature data.^15,20^ Swisher-McClure et al.^15^ obtained similar results in SGCs, with better dose-sparing of OARs with proton versus VMAT plans. Romesser et al.^20^ reported lower rates of grade >2 acute dysgeusia (5.6% vs 65.2%), mucositis (16.7% vs 52.2%), nausea (11.1% vs 56.5%), and fatigue (5.6% vs 8.7%), with an overall p-value < .05 in patients with salivary gland tumors treated with PT compared to IMRT.^15^ Clinical advantages with PT were also confirmed in a recent study by Dagan et al.,^21^ in which rates of acute toxicities were excellent, with most patients experiencing no greater than grade 1 mucositis, dysphagia, dysgeusia in a cohort of 23 patients treated both in the curative and adjuvant setting. Similarly, a recent study performed by Hanania et al.^22^ showed low rates of acute mucosal toxicity with PT in a cohort of 72 patients with major salivary gland cancer. Of note to that research, the majority of patients were treated with IMPT, while much of the previous works have been performed using a passively scattered PT. In this regard, the reduction of absorbed dose to different OARs provided by IMPT could translate into decreasing mucositis, odynophagia, swallowing dysfunction or aspiration with reduction of treatment breaks and less pain management therapy, particularly for patients with locally advanced diseases. Nevertheless, further analyses on larger cohort of patients with clinical measurable outcomes are required to support these encouraging dosimetric findings. Moreover, most of these few clinical comparison studies aimed to evaluate differences in terms of acute toxicities. However, the majority of patients with PGCs usually have longer follow-up and survival with respect to other HNCs and so there is a need to focus future analyses not only on acute but also on late radiation-related side effects.

In the present study, IMPT nearly eliminated the amount of dose to contralateral structures, such as the parotid and the submandibular glands, as compared to VMAT, while maintaining conformal target dose coverage. The benefit of sparing the contralateral parotid gland and submandibular gland is of particular importance in this setting of patients, as the function of their ipsilateral parotid gland has deteriorated. However, it remains uncertain whether a much lower dose to the major salivary glands would reduce the rate of xerostomia. Animal models have suggested that even low doses to the high-density stem cell regions of the salivary gland might impact on tissue function.^40^ Furthermore, Hanania et al.^22^ described low rates of late xerostomia in their cohort of patients, which may suggest a clinical benefit of the dose sparing. The median follow-up in that clinical study was 41 months, only available for 53 patients and none of them developed late toxicities higher than grade 3.^23^ However, further investigations and longer follow up are warranted to determine the impact of these dosimetric findings on late toxicity in real-life clinical practice. Other than sparing the major salivary glands, minor salivary glands, located all over the upper aerodigestive tract (e.g. oral cavity, buccal mucosa, pharynx), are responsible for basal salivation and general oral health and maintenance. Reducing Dmean to these structures could potentially translate into a clinical advantage.

Similar to the findings of the dosimetric study by Swisher-McClure et al.,^15^ in the current research, it was found that differences in Dmean to the brainstem are statistically significant between IMPT and VMAT. According to the literature, the Dmean to brainstem could eventually be related to radiation-induced nausea and vomiting. In this regard, Rosenthal et al.^41^ reported 76% and 38% of patients treated with IMRT without chemotherapy had nausea and vomiting, respectively. Wang et al.^42^ found that avoiding dose to the area postrema and the dorsal vagus complex may reduce both nausea and vomiting. Thanks to a better sparing of these structures PT might result in a reduction of nausea and vomiting related to radiation treatment.^43^

In our study IMPT also resulted in a statistically significant reduction of the Dmean to the ipsilateral cochlea when compared to VMAT, in contrast to a similar study recently published,^15^ where the IMPT-VMAT Dmeans difference to the ipsilateral inner ear (although in favor of IMPT) did not reach statistical significance. In this regard, we might point out that in the latter mentioned study it was not used the IMPT technique for the treatment planning PT calculation, thus possibly explaining the different results obtained.

Other than a dosimetric comparison, in our study a planning comparison based on SGCs-specific toxicities derived from clinical experience was also performed. In recent years, a model-based approach has been successfully employed in clinical settings, in particular for HNCs. However, due to the rarity of disease, patients with SGCs are generally under-represented in studies developing, validating or applying NTCP models. Therefore, our study can fill this void.

In our cohort, although limited to using only one NTCP model externally validated, following the model-based approach,^23^ ten patients (27%) qualified for PT based on expected reduction of hearing loss and tinnitus of grade 2 and eight (22%) qualified for PT based on NTCP for tinnitus. These results could pave the way for the use of NTCP models for PGCs, particularly for young and good prognosis patients.

None of the patients in our study had an advantage in terms of reduction in the anticipated probability of trismus, dysphagia, dysgeusia and acute oral mucositis. This finding is not surprising since these endpoints generally do not represent relevant toxicities in patients treated for PGCs compared to tumors located elsewhere (for example nasopharyngeal cancer).

Is it noteworthy that in our study patients with microscopic or macroscopic perineural tumor invasion were excluded, since in these cases the CTV volume should encompass the inner ear as a route of tumor spread in such cohort of patients. On one hand, this allowed for more homogenous target volumes delineation, on the other this choice prevented us from including more potential PT candidates according to other toxicity-related endpoints such as those to neurological structures (temporal lobe and/or brainstem) or optic pathways.^39,44,45^

We are aware that our study has some pitfalls. Among the limitation of this retrospective work - other than the small sample size - we acknowledge that only one among the implemented NTCP models was developed on patients treated with PT. Indeed, the majority of NTCP models were developed in photon-based IMRT settings without taking into account the peculiar physical and radiobiological properties of PT.^46,47^ Moreover, due to the retrospective nature of the present analysis, the availability of qualitative toxicity data represented a limiting factor for the implementation of NTCP models which included clinical and/or functional parameters. Indeed, it is relevant to keep in mind the need to integrate dosimetric features with clinical characteristics, in order to build multivariable NTCP models.^37^ Furthermore, the majority of the considered NTCP models were developed in a cohort of patients with different subsites of HNCs with respect to our population, mainly due to the fact that SGCs represent a rare disease among HNCs and are not commonly included in the development of NTCP models. Moreover, in the era of personalization, the intrinsic radiosensitivity of patients and its role in radiation treatment toxicities should be considered; Deneuve et al. recently published a study exploring the combination of radiosensitivity biomarker and NTCP models, demonstrating a better performance in predicting acute toxicities in HNCs patients than NTCP models alone.^48^

Among the strengths of this study, we might mention that the contouring of OARs in both plans (VMAT and IMPT) was performed by the same radiation oncologist in order to avoid the risk of inter-observer variability that could affect the quality of NTCP-based analysis. The calculation of NTCP values was automated by an in-house developed Python script implemented on the TPS, which minimizes errors in the extraction and collection of dosimetric parameters otherwise potentially inherent to the use of external devices. Finally, our study is focused on PGCs only, which, as rare diseases, are usually not comprehended in NTCP model-based literature. Therefore, we believe that the results of our work might provide an original and reliable setting of dosimetric data which could not only provide support to already available clinical findings reported in the literature, but could also be the basis for further analysis on the topic.

## Conclusions

In the current study, nearly one-third of the patients of our cohort were eligible for PT (according to NIPP criteria using available NTCP models), as the most likely to benefit from IMPT in terms of prevention of hearing loss and tinnitus. Moreover, a dosimetric comparison demonstrated the advantage of IMPT over VMAT plans, with comparable target coverage, for the majority of the OARs.

Finally, the present study not only provides a dosimetric support to implement the use of NTCP models also in the setting of PGCs but could represent a benchmark to investigate further models for peculiar treatment-related toxicities occurring in the parotid gland region (such as mastoiditis and osteoradionecrosis of the jaw).

## Supplemental Material

sj-docx-1-tmj-10.1177_03008916241252544 – Supplemental material for Dosimetric and NTCP analyses for selecting parotid gland cancer patients for proton therapy Supplemental material, sj-docx-1-tmj-10.1177_03008916241252544 for Dosimetric and NTCP analyses for selecting parotid gland cancer patients for proton therapy by Anna Maria Camarda, Maria Giulia Vincini, Stefania Russo, Stefania Comi, Francesca Emiro, Alessia Bazani, Rossana Ingargiola, Barbara Vischioni, Stefania Volpe, Roberto Orecchia, Barbara Alicja Jereczek-Fossa, Ester Orlandi and Daniela Alterio in Tumori Journal
