# Supplementary material for: Dosimetric and NTCP analyses for selecting parotid gland cancer patients for proton therapy
Source: Tumori. 2024 May 21;110(4):273–83. doi: 10.1177/03008916241252544 (PMC11295422; doi:10.1177/03008916241252544)
Supplement: sj-docx-1-tmj-10.1177_03008916241252544 – Supplemental material for Dosimetric and NTCP analyses for selecting parotid gland cancer patients for proton therapy [file sj-docx-1-tmj-10.1177_03008916241252544.docx]

**Supplementary Materials**

**Table S1.** Details of chosen normal tissue complication probability models.

| **Author,**  **year** | **Organ/toxicity** | **RT technique** | **MODEL** | **Variables included in the final model** | **Overall quality** |
| --- | --- | --- | --- | --- | --- |
| Christianen 2012  [31] | Physician-rated swallowing dysfunction 6 months after (CH) RT | 3DCRT  (62%),  IMRT  (38%) | Logistic regression  NTCP = (1 + e^-s)^-1,  where s= -6.09 + (Dmean SPC x 0.057) + (Dmean supraglottic larynx) | Dmean superior PCM  Dmean supraglottic larynx. | Good  - Endpoint >=12m from RT start: no  - Advanced RT techniques only, no reirradiation: no  - N pts. / N events: 354/ NA  - Validation: yes (external)  - Prediction probability: AUC 0.80 |
| Lindblom, 2014  [32] | Trismus:  MID  <35mm after 21-  127m (median  66 m) post RT start  and problems  mouth-opening  (EORTC H&N35  QoL scores of  3–4) at several  TPs until 5y post  RT-start | 3DCRT  (95%),  IMRT  (5%) | Logistic regression  NTCP = 1 / (1 + e^4γ^(1 – D/D50))  Model parameters for masseter:  MID: TD50=72.3 Gy, y50=1.04  QoL scores: TD50=57.2 Gy, y50=0.78 | Dmean to the ipsilateral masseter for both endpoints | Good  - Endpoint >=12m from RT start: yes for MID, unclear for QoL scores  - Advanced RT techniques only, no reirradiation: no  - N pts. / N events: 121/50  - Validation: yes (internal cross validation)  - Prediction probability: 0.77 and 0.73 for model with endpoint MID and QoL score 3+, respectively and ipsi masseter included |
| Sapir, 2016 [33] | Severe  dysgeusia (UWQOL and  HNQOL  questionnaire,  score >50/100) at 3m post-RT | IMRT | LKB  HNQOL: D50 = 53 Gy, m=0.4, n=unclear;  UWQOL: D50 = 57 Gy, m=0.4, n=unclear | Dmean oral cavity | Fair  - Endpoint >=12m from RT start: no  - Advanced RT techniques only, no reirradiation: yes  - N pts. / N events: 73/26  - Validation: no |
| De Marzi, 2015  [34] | Hearing loss  >15 dB at two  contiguous test  frequencies  >6mo post-RT | PT | LKB (gEUD)  Inner ear  TD50=53.7 Gy, y=2.8, a=0.1 | Dmean inner ear | Fair  - Endpoint >=12m from RT start: yes (median FU 26 months)  - Advanced RT techniques only: yes  - N ears/ N events: 280/73  - Validation: no  - Prediction probability: AUC 0.86 |
| Lee, 2015  [35] | G2+ tinnitus  (LENT-SOMA)  between 36 –  77m post-RT | IMRT | LKB and Logistic regression  NTCP = exp(4y50 x (MD/TD50 – 1))/1+ exp  (4y50 x (MD / TD50 – 1)),  where “MD is the mean dose to the  cochlea” and “y50 is the normalized slope  of the dose-response curve”  LKB:  TD50=46.52Gy, m=0.35  Logistic: TD50=46.31Gy, y50=1.27 | Dmean cochlea ipsilateral | Fair  - Endpoint >=12m from RT start: yes  - Advanced RT techniques only, no reirradiation: yes  - N inner ears / N events: 422/49  - Validation: no  - Prediction probability: LKB: 0.76, Logistic: 0.76  - Others: influence of chemotherapy not accounted for |
| Orlandi, 2018  [36] | Acute oral mucositis  G > 1.5 | IMRT | Lasso Logistic regression  NTCP = (1 + e^-s)^-1,  where s= - 4.1453 + 0.0699(EUD) N=1 | Dmean Oral cavity | Fair  - Endpoint >=12m from RT start  - Advanced RT techniques: IMRT, no reirradiation  - N pts. /N events: 132/40  - Validation: no |

**Abbreviations:** 3DCRT: 3D conformal radiotherapy; AUC: area under the curve; CH: chemotherapy; Dmean: mean dose; EUD: equivalent uniform dose; G: grade; IMPT: intensity modulated proton therapy; IMRT: intensity modulated radiotherapy; LKB: Lyman Kutcher Burman; MID: Maximal interincisal distance; PCM: pharyngeal constrictor muscles; PT: proton therapy; QoL: quality of life; RT: radiotherapy; TP: time point.

**Table S2**. Average and median clinical target volume coverage parameters

|  |  | **Average** | **Median** |
| --- | --- | --- | --- |
| **CTV D99 (Gy)** | VMAT | 61.53 | 62.85 |
|  | IMPT | 63.98 | 64.62 |
|  | p value *(VMAT vs. IMPT)* | <.00001 | |
| **CTV D98 (Gy)** | VMAT | 62.62 | 64.15 |
|  | IMPT | 64.4 | 64.96 |
|  | p value *(VMAT vs. IMPT)* | <.00001 | |
| **CTV D95 (Gy)** | VMAT | 63.92 | 65.11 |
|  | IMPT | 64.94 | 65.38 |
|  | p value *(VMAT vs. IMPT)* | <.00001 | |
| **CTV D2 (Gy)** | VMAT | 67.92 | 68.23 |
|  | IMPT | 67.01 | 67.35 |
|  | p value *(VMAT vs. IMPT)* | <.00001 | |
| **CTV D1 (Gy)** | VMAT | 68.16 | 68.44 |
|  | IMPT | 67.17 | 67.48 |
|  | p value *(VMAT vs. IMPT)* | <.00001 | |

***Abbreviations:*** *CTV: Clinical Target Volume; VMAT: Volumetric Modulated Arc Therapy; IMPT: Intensity Modulated Proton Therapy.*

**Table S3.** NTCP values and ΔNTCP_x-p_ in terms of hearing loss and tinnitus for single patients.

|  | **NTCP Hearing loss** | | **ΔNTCP_x-p_** | **NTCP Tinnitus** | | **ΔNTCP_x-p_** |
| --- | --- | --- | --- | --- | --- | --- |
| **ID** | **VMAT** | **IMPT** |  | **VMAT** | **IMPT** |  |
| 1 | 0 | 0 | 0.00 | 0.01 | 0 | 0.01 |
| 2 | 0 | 0 | 0.00 | 0.01 | 0 | 0.01 |
| 3 | 0 | 0.72 | -0.72 | 0.06 | 0.59 | -0.53 |
| 4 | 0.99 | 0.99 | 0.00 | 0.46 | 0.57 | -0.11 |
| 5 | 0.00 | 0.03 | -0.03 | 0.06 | 0.26 | -0.20 |
| 6 | 0.00 | 0.00 | 0.00 | 0.09 | 0.09 | 0.00 |
| 7 | 0.00 | 0.00 | 0.00 | 0.05 | 0.11 | -0.06 |
| **8** | 0.42 | 0.21 | **0.21** | 0.70 | 0.58 | **0.12** |
| 9 | 0.03 | 0.04 | -0.01 | 0.47 | 0.53 | -0.06 |
| **10** | 0.69 | 0.44 | **0.25** | 0.77 | 0.65 | **0.12** |
| **11** | 0.17 | 0.06 | **0.11** | 0.52 | 0.40 | **0.12** |
| 12 | 0.44 | 0.57 | -0.13 | 0.57 | 0.72 | -0.15 |
| **13** | 0.29 | 0.18 | **0.11** | 0.68 | 0.58 | **0.10** |
| 14 | 0.06 | 0.19 | -0.13 | 0.40 | 0.55 | -0.15 |
| **15** | 0.69 | 0.47 | **0.22** | 0.77 | 0.66 | **0.11** |
| **16** | 0.72 | 0.53 | **0.19** | 0.77 | 0.69 | 0.08 |
| **17** | 0.92 | 0.58 | **0.34** | 0.87 | 0.69 | **0.18** |
| 18 | 0.03 | 0.06 | -0.03 | 0.29 | 0.45 | -0.16 |
| **19** | 0.07 | 0.00 | 0.07 | 0.31 | 0.09 | **0.22** |
| 20 | 0.00 | 0.00 | 0.00 | 0.01 | 0.01 | 0.00 |
| 21 | 0.00 | 0.00 | 0.00 | 0.00 | 0.00 | 0.00 |
| 22 | 0.00 | 0.00 | 0.00 | 0.03 | 0.01 | 0.02 |
| 23 | 0.00 | 0.00 | 0.00 | 0.01 | 0.00 | 0.01 |
| 24 | 0.00 | 0.00 | 0.00 | 0.03 | 0.01 | 0.02 |
| 25 | 0.00 | 0.00 | 0.00 | 0.05 | 0.02 | 0.03 |
| 26 | 0.00 | 0.00 | 0.00 | 0.03 | 0.01 | 0.02 |
| **27** | 0.17 | 0.05 | **0.12** | 0.20 | 0.22 | -0.02 |
| 28 | 0.00 | 0.00 | 0.00 | 0.06 | 0.01 | 0.05 |
| 29 | 0.00 | 0.00 | 0.00 | 0.07 | 0.02 | 0.05 |
| 30 | 0.00 | 0.00 | 0.00 | 0.08 | 0.02 | 0.06 |
| 31 | 0.00 | 0.00 | 0.00 | 0.02 | 0.01 | 0.01 |
| **32** | 0.25 | 0.02 | **0.23** | 0.33 | 0.19 | **0.14** |
| 33 | 0.00 | 0.00 | 0.00 | 0.02 | 0.00 | 0.02 |
| 34 | 0.00 | 0.00 | 0.00 | 0.01 | 0.00 | 0.01 |
| 35 | 0.00 | 0.00 | 0.00 | 0.01 | 0.00 | 0.01 |
| 36 | 0.00 | 0.00 | 0.00 | 0.01 | 0.00 | 0.01 |
| 37 | 0.00 | 0.00 | 0.00 | 0.15 | 0.17 | -0.02 |
| **Median** | 0.00 | 0.00 | 0.00 | 0.07 | 0.09 | 0.01 |
| **Mean** | 0.16 | 0.14 | 0.02 | 0.24 | 0.24 | 0.00 |
